# A Simple Three-Step Method for the Synthesis of Submicron Gold Particles: The Influence of Laser Irradiation Duration, Pulse Energy, Laser Pulse Duration, and Initial Concentration of Nanoparticles in the Colloid

**DOI:** 10.3390/nano16020079

**Published:** 2026-01-06

**Authors:** Ilya V. Baimler, Ivan A. Popov, Alexander V. Simakin, Sergey V. Gudkov

**Affiliations:** 1 Prokhorov General Physics Institute of the Russian Academy of Sciences, 38 Vavilova St., 119991 Moscow, Russia; ilyabaymler@yandex.ru (I.V.B.); popovia2003@yandex.ru (I.A.P.); avsimakin@gmail.com (A.V.S.); 2 Department of Fundamental Sciences, Bauman Moscow State Technical University, 5 2nd Baumanskaya St., 105005 Moscow, Russia

**Keywords:** laser ablation, laser fragmentation, laser melting, gold nanoparticles

## Abstract

This work demonstrates a three-step method for the synthesis and production of submicron spherical gold particles using laser ablation in liquid (LAL), laser-induced fragmentation in liquid (LFL), laser-induced nanochain formation, and laser melting in liquid (LML). The nanoparticles were characterized using transmission electron microscopy (TEM), dynamic light scattering (DLS), and UV–visible spectroscopy. In the first stage, spherical gold nanoparticles with a size of 20 nm were obtained using LAL and LFL. Subsequent irradiation of gold nanoparticle colloids with radiation at a wavelength of 532 nm leads to the formation of gold nanochains. Irradiation of nanochain colloids with radiation at a wavelength of 1064 nm leads to the formation of large spherical gold particles with a size of 50 to 200 nm. The formation of submicron gold particles upon irradiation of 2 mL of colloid occurs within the first minutes of irradiation and is complete after 480,000 laser pulses. Increasing the laser pulse energy leads to the formation of larger particles; after exceeding the threshold energy (321 mJ/cm^2^), fragmentation is observed. Increasing the concentration of nanoparticles in the initial colloid up to 150 μg/mL leads to a linear increase in the size of submicron nanoparticles. The use of picosecond pulses for irradiating nanochains demonstrates the formation of the largest particles (200 nm) compared to nanosecond pulses, which may be due to the effect of local surface melting. The described technique opens the possibility of synthesizing stable gold nanoparticles over a wide range of sizes, from a few to hundreds of nanometers, without the use of chemical reagents.

## 1. Introduction

In recent years, laser ablation in liquid has been one of the main methods for synthesizing various nanoparticles [1]. The capabilities of LAL are constantly expanding, demonstrating a steady increase in productivity [2,3], an expansion of the range of synthesized materials [4,5] and an increase in the areas of application of nanoparticles, for example, biomedicine [6,7], catalysis [8,9], energy storage [10,11,12,13], electronics [14,15], etc.

Nanoparticle size control in laser nanoparticle synthesis is an important aspect of the synthesis process, since various properties of nanoparticles are largely determined by their sizes [16]. Laser irradiation of nanoparticle colloids is a well-known method for modifying nanoparticle sizes, in which larger nanoparticles are fragmented by laser radiation to form smaller nanoparticles. The resulting size of the smaller particles can be controlled by varying the radiation parameters [17,18]. It is known that the reduction in particle size can be caused by evaporation, phase explosion of nanoparticles, or Coulomb explosion caused by the emission of electrons from metallic nanoparticles, followed by fission due to electrostatic repulsion [19,20].

Nanoparticle shape modification is often achieved using laser melting in liquid techniques [21,22,23]. Laser melting of metallic nanoparticles has been used in laser nanotechnology for spheroidization of initially non-spherical NPs [24]. Generally, two types of nanoparticle melting are distinguished: localized surface melting of nanoparticles, which is achieved using ultrashort (femto- and pico-) laser pulses [25], and complete melting of nanoparticles, which is usually observed using nanosecond pulses [26]. Potentially, sequential melting and subsequent aggregation of molten nanoparticles could be used to circumvent the size limitations of laser ablation and laser fragmentation methods.

However, only a few studies have described the fusion of nanoparticles into larger nanostructures as a result of laser irradiation of nanoparticle colloids. It is still unclear how this process can be controlled [27,28]. One of the shortcomings of current studies devoted to the synthesis of large particles from small ones by laser melting is the use of third-party reagents to initiate the aggregation and stabilization of nanoparticles. This deprives one of the significant advantages of laser ablation, laser fragmentation, and laser melting methods—the ability to obtain nanoparticles without the use of toxic and environmentally hazardous reagents. The absence of reagents in the nanoparticle colloid is an advantage when using particles in biotechnology [29], the synthesis of new materials [30], as substrates for surface Raman spectroscopy [31] in surface-activated laser desorption and ionization mass spectrometry [32], since third-party reagents create additional signals that often interfere with detection. However, one of the potential disadvantages of using only laser methods for particle synthesis may be the low yield of submicron particles compared to chemical methods. Currently, one of the main strategies for increasing the production of nanoparticles is the optimization of laser radiation parameters (laser fluence, wavelength, repetition rate, pulse duration) during the synthesis process [33,34] and new experiment configurations [35,36], allowing the productivity increase of particle synthesis using laser radiation. Recent studies show that it is possible to further increase production rate of nanoparticles during LAL by application of stirring and sonication techniques [37,38]. The use of these methods allows for the effective removal of cavitation bubbles or acceleration of their collapse, thereby eliminating radiation scattering and increasing the efficiency of laser energy absorption by the bulk material. Similar strategies can be applied to laser melting of nanoparticles to increase the efficiency of submicron particle formation.

This study demonstrates for the first time a three-step method for synthesizing submicron gold particles without the use of external reagents and substances. The method includes the following steps: 1. synthesis of spherical gold nanoparticles using laser ablation and fragmentation; 2. formation of elongated nanochains and aggregates from gold particles; 3. melting of elongated nanochain colloids, resulting in the formation of submicron gold particles. The influence of laser irradiation duration, pulse energy, laser pulse duration and initial concentration of nanoparticles in the colloid on the final result of synthesis was studied. As a result, the optimal parameters were established for the most efficient synthesis of submicron particles. The method described in the work makes it possible to obtain spherical gold particles of submicron size, which in turn can be further used as cell markers [39], light scattering media [40], biosensors [41] and drug-delivery carriers [42]. Such particles can be used to create SERS labels and can serve as effective labels for confocal laser microscopy [43]. In addition, it is possible to use submicron particles in biomedicine due to their lower toxicity compared to smaller nanoparticles [44,45].

## 2. Materials and Methods

### 2.1. The Stage of Obtaining Spherical Gold Nanoparticles AuNPs(I) Using Laser Ablation and Laser Fragmentation Techniques in Liquid

Spherical AuNPs(I) nanoparticles were synthesized by laser ablation and fragmentation of a solid Au target (99.99%) in deionized water (18 MΩ × cm). A classical experimental setup for laser ablation in liquid was used in the experiment. The gold target was attached to the bottom of a 30 mL glass cuvette and filled with deionized water. The thickness of the liquid layer between the target surface and the liquid was 2–3 mm. A pulsed Nd:YAG laser NL300 (Ekspla, Vilnius, Lithuania) with a laser pulse duration of 4 ns, a repetition rate of 1 kHz, a wavelength of 1064 nm, and a pulse energy of 2 mJ was used as a laser source. The laser spot size at the waist was 200 μm. Using an LScanH galvanomechanical scanner (Ateko-TM, Moscow, Russia) and an F-Theta objective with a focal length of 90 mm, a focused radiation beam was moved along the surface of a gold target. The laser beam trajectory consisted of several parallel lines inscribed in a 1 × 1 cm square. The line density was 70 lines per millimeter. The beam scanning speed using the scanner system was 3000 mm/s. The selected parameters for line density and laser beam speed were determined based on considerations of maximizing nanoparticle formation yield, which in turn requires each subsequent laser pulse to hit a target location free of cavitation bubbles from previous pulses [46]. Optimal parameters were established by measuring the mass concentration of nanoparticles in preliminary experiments. The ablation time was 30 min. This laser ablation procedure resulted in gold nanoparticle colloids with a nanoparticle mass concentration of 100 μg/mL, which was determined by weighing the gold target before and after laser ablation using an OHAUS Pioneer PA114C analytical laboratory balance (Parsippany, NJ, USA). Different concentrations of nanoparticles in the colloid were obtained by varying the duration of laser ablation.

The colloidal solution of gold nanoparticles obtained by laser ablation was subjected to repeated irradiation—laser fragmentation. For this purpose, the resulting colloid of gold nanoparticles was placed in a glass cuvette with a transparent bottom. Using reflective mirrors, the beam was directed into the cuvette from below, through the transparent bottom, and focused using an F-Theta objective at a distance of 1–2 cm from the cuvette bottom. During colloid fragmentation, the beam was also moved within the colloid using a scanning system with the same parameters for filling density and movement speed (line density—70 lines/ mm, beam scanning speed—3000 mm/s). The fragmentation time of the gold nanoparticle colloid was 30 min. Using this method, a colloid of AuNPs(I) nanoparticles was obtained, which was considered as the initial.

### 2.2. The Stage of Obtaining Elongated Gold Nanochains AuNPs(II)

AuNPs(II) samples, representing elongated gold nanochains, were obtained by irradiating the initial 30 mL colloid of spherical AuNPs(I) nanoparticles with laser radiation at the second harmonic of an NL300 Nd:YAG laser with the following parameters: pulse duration of 4 ns, repetition rate of 1 kHz, wavelength of 532 nm, pulse energy of 1 mJ. The laser spot size at the beam waist was 300 μm. The irradiation duration was 10 min. The same laser beam movement system was used for irradiation as in the case of laser fragmentation of nanoparticles. The described irradiation technique was used to obtain a colloid of AuNPs(II) gold nanoparticles.

### 2.3. The Stage of Obtaining Submicron Gold Particles AuNPs(III)

AuNPs(III) samples were obtained by repeated irradiation of colloids of elongated AuNPs(II) gold nanochains using Nd:YAG laser radiation at the first harmonic of an NL300. A 2 mL cuvette was used for experiments to obtain large AuNPs(III) gold particle samples.

**Figure 1:**
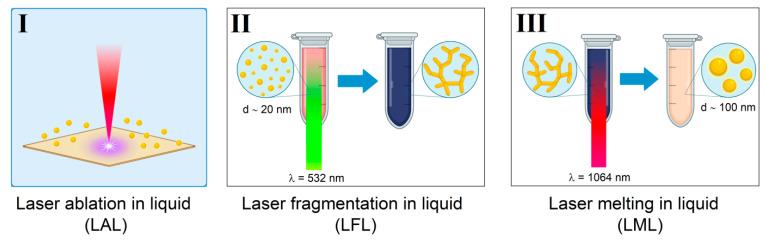
Schematic representation of a three-stage process for producing submicron gold particles: I—stage of laser ablation and synthesis of spherical gold nanoparticles; II—stage of irradiation of a colloid of gold nanoparticles with the second harmonic radiation of an Nd:YAG laser with the formation of gold nanochains; III—stage of laser melting of nanochains and the formation of submicron spherical gold particles.

To study the dependence of gold nanoparticle morphology on the laser pulse duration, Nd:YAG lasers PL PDP-3114SH (wavelength—1064 nm, pulse duration—30 ps, pulse repetition rate—1 kHz, pulse energy—50 μJ, laser spot size at the waist—200 μm) and P-Mark TT 100 (wavelength—1064 nm, pulse duration—200 ns, pulse repetition rate—20 kHz, pulse energy—50 μJ, laser spot size at the waist—200 μm) were used as laser radiation sources. Schematically, all stages of obtaining submicron gold particles are shown in Figure 1.

### 2.4. Morphology Analysis of Gold Nanoparticles

To study the size distribution of nanoparticles and determine the concentration of nanoparticles, a Zetasizer Ultra RedLebel 10 particle analyzer (Malvern Panalytical, Malvern, UK) based on the dynamic light scattering technique was used.

A Libra 200FE HR transmission electron microscope (TEM) (Carl Zeiss, Jena, Germany) was used to image Au nanoparticles and study their morphology. Copper microscopic grids were used to prepare the Au nanoparticles for TEM analysis.

The absorption spectra of colloidal gold nanoparticle solutions were measured using a USB3000T spectrometer (Ocean Optics, Orlando, FL, USA) (200–800 nm). Spectra were measured in 2 mL quartz cuvettes with an optical path length of 1 cm. The absorption spectra of deionized water used for laser ablation and fragmentation were used as reference spectra. The spectra were normalized to a wavelength of 400 nm.

## 3. Results and Discussion

### 3.1. Morphology and Characteristics of Obtained AuNPs

**Figure 2:**
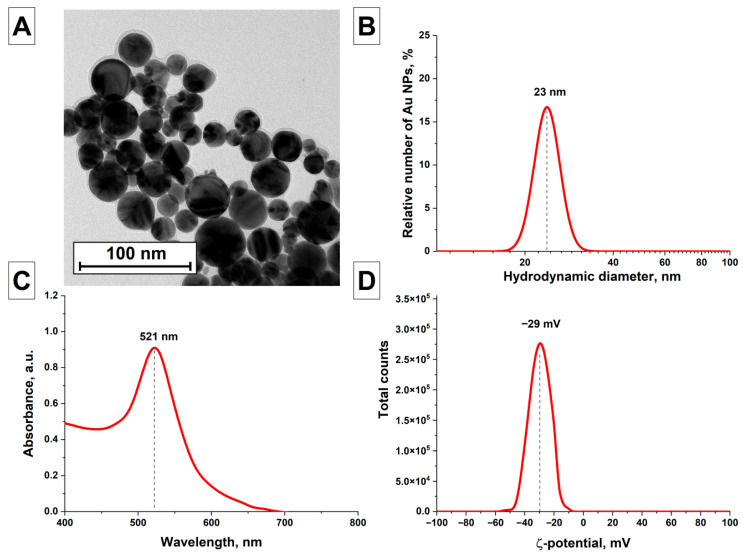
Characterization of the AuNPs(I) obtained by laser ablation in water; (**A**) TEM image of AuNPs(I) nanoparticles (scale bar is 100 nm); (**B**) Distribution of hydrodynamic diameters of AuNPs(I) obtained by DLS technique; (**C**) Absorption spectrum of aqueous colloid of AuNPs(I) nanoparticles; (**D**) Distribution of ζ-potential of AuNPs(I) nanoparticles.

Figure 2 shows a TEM image of spherical gold nanoparticles AuNPs(I) obtained as a result of laser ablation and subsequent laser fragmentation of the colloid in water (Figure 2A). It is shown that the gold nanoparticles have a spherical shape, the sizes of the nanoparticles range from 10 to 30 nm. Figure 2B shows the results of the analysis of the nanoparticle colloid using the DLS technique. It was found that the distribution of gold nanoparticles depending on their hydrodynamic size has a monomodal shape. The maximum of the particle distribution is at a size of 23 nm. The half-width of the distribution at half-maximum is 6 nm. Figure 2C shows the absorption spectrum of an aqueous colloid of gold nanoparticles AuNPs(I). The presented absorption spectrum of a gold nanoparticle colloid demonstrates enhanced absorption, which corresponds to the surface plasmon resonance of gold nanoparticles. This resonance arises from collective oscillations of free electrons in the conduction band from one surface of the material nanoparticle to another during the interaction of electrons with electromagnetic radiation [47]. The position of the SPR peak depends on the size, shape, and concentration of the nanoparticles [48,49]. Furthermore, colloids of elongated nanoparticles exhibit enhanced absorption in the red region due to longitudinal oscillations of electrons occurring in the material, which shift the SPR maximum toward longer wavelengths. The absorption maximum, located at a wavelength of 521 nm, and the absence of absorption in the red region of the spectrum indicate the presence of only spherical gold nanoparticles in the colloid. According to previous studies, the particle sizes can be estimated at 10–20 nm. [50]. The distribution of the electrokinetic potential of gold nanoparticles in a colloid was obtained (Figure 2D). It was shown that the maximum of the zeta potential distribution in the colloid is at −29 mV, which typically indicates good particle stability in the colloid.

**Figure 3:**
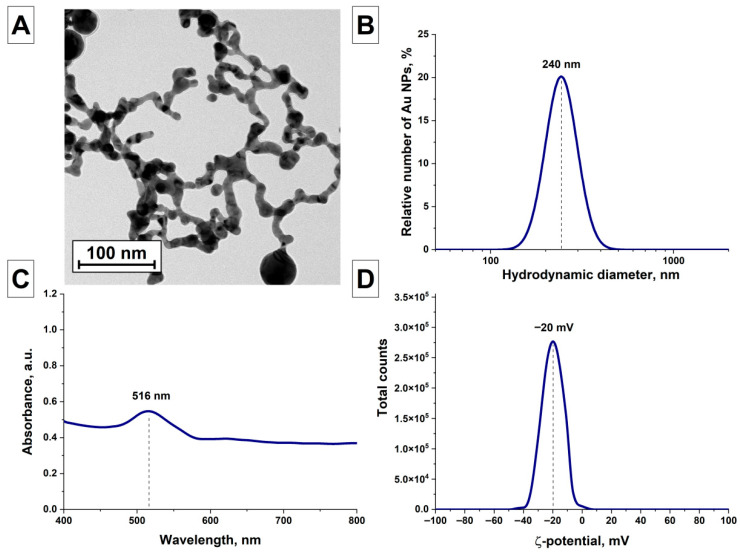
Characteristics of AuNPs(II) nanoparticles obtained after irradiation of the initial colloid at a wavelength of 532 nm; (**A**) TEM image of the obtained AuNPs(II) nanoparticles (scale bar is 100 nm); (**B**) Distribution of AuNPs(II) nanoparticles depending on the hydrodynamic diameter; (**C**) Absorption spectrum of an aqueous colloidal solution of AuNPs(II) nanoparticles; (**D**) Distribution of ζ-potential of AuNPs(II) nanoparticles.

The morphological properties of AuNPs(II) gold nanoparticles obtained by irradiating the initial AuNPs(I) nanoparticles sample with laser radiation at the second harmonic of an Nd:YAG laser were studied, Figure 3. Figure 3A shows a TEM image of AuNPs(II) gold nanochains, which are elongated and melted together spherical nanoparticles connected by bridges several tens of nanometers long and 10–20 nm thick. Analysis of the obtained sample using the DLS technique shows that the size distribution of the obtained particles is monomodal. The distribution maximum is at a size of 240 nm. The half-width of the distribution is 120 nm, Figure 3B. The absorption spectrum of the AuNPs(II) gold nanoparticles colloid was studied, Figure 3C. The absorption spectrum shows a peak of plasmon resonance of gold nanoparticles at a wavelength of 516 nm. The absorption spectrum of the colloid also exhibits absorption in the red region, indicating the presence of elongated gold nanoparticles with a large longitudinal-to-transverse aspect ratio, as confirmed by TEM images. The zeta potential of the AuNPs(II) nanoparticle colloid was studied (Figure 3D). It was found that the maximum of the electrokinetic potential distribution is located at −20 mV, indicating lower stability of the AuNPs(II) nanoparticles in the colloid and their greater tendency to form aggregates than in the original AuNPs(I) nanoparticle colloid.

**Figure 4:**
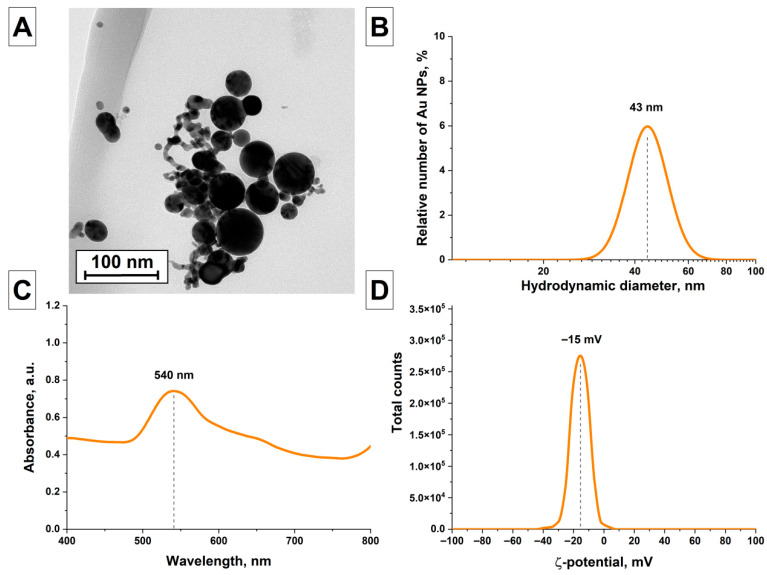
Characterization of AuNPs(III) particles obtained after irradiation of the colloid at a wavelength of 1064 nm; (**A**) TEM image of the obtained AuNPs(III) particles (scale bar is 100 nm); (**B**) Size distribution of AuNPs(III) particles obtained by DLS method; (**C**) Absorption spectrum of an aqueous colloid of AuNPs(III) particles; (**D**) Distribution of ζ-potential of AuNPs(III) particles.

The characteristics of gold particles AuNPs(III) obtained by irradiating AuNPs(II) samples with Nd:YAG laser radiation at the first harmonic with a sufficiently low pulse energy (units and tens of μJ) were studied, Figure 4. It was shown that as a result of irradiation of elongated gold nanochains at low pulse energies, both nanochains and larger spherical particles are present in the colloid, which is shown in the TEM image, Figure 4A. At the same time, in the particle size distribution obtained using a particle size analyzer, a shift in the particle distribution towards larger sizes is observed, Figure 4B. It is shown that the distribution has a monomodal shape with a maximum located at 43 nm and a distribution half-width of 16 nm. The absorption spectrum of the AuNPs(III) sample was studied, Figure 4C. The absorption spectrum is shown to contain a maximum located at 540 nm, with absorption also observed in the longer wavelength region. A shift of the absorption maximum toward longer wavelengths indicates an increase in the size of the particles in the colloid; in addition, the colloid also contains elongated particles. The electrokinetic potential distribution with a maximum at −15 mV indicates the low stability of the colloid (Figure 4D).

### 3.2. The Influence of Irradiation Time on the Spectral Characteristics of Gold NPs

**Figure 5:**
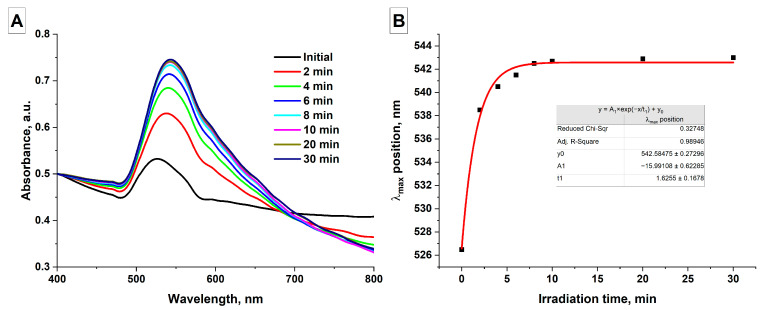
Effect of laser irradiation duration; (**A**) Normalized absorption spectra of aqueous colloids of gold nanoparticles depending on irradiation time (λ = 1064 nm, ε = 30 μJ, energy density—98 mJ/cm^2^); (**B**) Position of the plasmon resonance peak in the absorption spectra of aqueous colloids of gold nanoparticles depending on irradiation duration. The red line represents the data fit.

The effect of laser irradiation duration on the optical absorption spectra of AuNPs(II) colloids was studied. Samples of elongated gold nanoparticles (AuNPs(II)) with a concentration of 50 μg/mL and a volume of 2 mL were irradiated with a Nd:YAG laser (wavelength 1064 nm, pulse duration 4 ns, pulse repetition rate 1 kHz, pulse energy 30 μJ, energy density 98 mJ/cm^2^) for varying periods of time. The absorption spectra of AuNPs(III) colloids were obtained as a result of the study (Figure 5).

It was shown that with increasing colloid irradiation time, the amplitude of the plasmon resonance peak in the absorption spectra increases. At the same time, a gradual shift of the absorption maximum toward longer wavelengths occurs, and a decrease in absorption in the red region of the spectrum is also observed in the colloids (Figure 5A). These changes in the absorption spectrum of gold nanoparticle colloids indicate the formation of larger spherical particles (an increase in the amplitude and a shift of the plasmon resonance peak toward the red region) [51,52], as well as the disappearance of elongated gold nanochains (a decrease in absorption in the red region of the spectrum) [53]. Thus, it can be assumed that under the influence of laser radiation, a change in the shape of gold nanochains occurs, namely, their melting, followed by the formation of larger spherical particles from the molten liquid material. It follows from Figure 5A that after irradiation of nanoparticle colloids for 8 min or more, no significant changes in the absorption spectrum are observed. This indicates the completion of the process of formation of larger gold particles from elongated nanochains in the colloid, due to the increasing scattering of radiation on large spherical gold particles, and the absence of absorption centers and the formation of molten material, i.e., gold nanochains. Figure 5B shows the position of the plasmon resonance peak in the absorption spectra as a function of the colloid irradiation time. It is shown that the initial position of the resonance peak corresponded to 527 nm in the AuNPs(II) nanoparticle sample. With increasing colloid irradiation time, the plasmon resonance peak shifts toward longer wavelengths and is already at 538 nm after 2 min of irradiation. Irradiation of a colloid of gold nanoparticles for 8–10 min leads to a shift in the absorption maximum to 542 nm; further irradiation of the colloid for 20 and 30 min does not lead to a change in the position of the peak. The process of melting elongated gold nanochains and synthesizing submicron gold particles from them when irradiating 2 mL of colloid with radiation with an energy density of approximately 100 mJ/cm^2^ is finished after approximately 480,000 laser pulses. Therefore, in subsequent experiments investigating the influence of other parameters on the process of gold nanoparticle formation, the irradiation of colloids was carried out for 8 min or with an equivalent number of laser pulses.

### 3.3. Effect of Laser Pulse Energy on the Spectral Characteristics of AuNPs(III)

**Figure 6:**
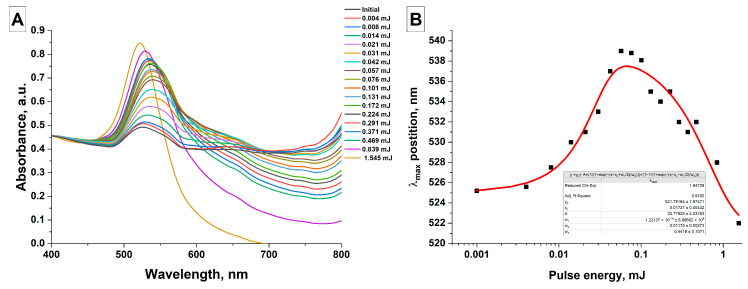
Effect of laser pulse energy; (**A**) Normalized absorption spectra of aqueous colloids of gold nanoparticles as a function of laser pulse energy (λ = 1064 nm, t = 8 min); (**B**) Position of the plasmon resonance absorption peak in the absorption spectra of aqueous colloids of gold nanoparticles as a function of pulse energy. The red line represents the data fit.

The influence of laser pulse energy on the absorption spectra of AuNPs(II) colloids after exposure to laser radiation from a Nd:YAG laser (wavelength—1064 nm, pulse duration—4 ns, pulse repetition frequency—1 kHz) on a 2 mL AuNPs(II) sample for 8 min was studied, Figure 6.

Figure 6A shows the dynamics of changes in the absorption spectra of gold nanoparticle colloids with an increase in laser pulse energy from 4 μJ to 1.5 mJ and, accordingly, an energy density of 12 mJ/cm^2^ to 4.9 J/cm^2^. It was found that irradiation of colloids of elongated gold nanoparticles AuNPs(II) in the pulse energy range from 4 μJ (12 mJ/cm^2^) to 57 μJ (181 mJ/cm^2^) leads to an increase in the plasmon resonance amplitude, a shift in the maximum position toward longer wavelengths and a decrease in absorption in the red region of the spectrum, which corresponds to the process of remelting elongated nanoparticles with each other and the formation of spherical particles larger than in the original AuNPs(I) colloid. With a further increase in the pulse energy from 76 μJ (242 mJ/cm^2^) to 1.5 mJ (4.9 J/cm^2^), a further increase in the plasmon resonance amplitude and a decrease in absorption in the long-wavelength region of the spectrum are observed. However, the absorption maximum begins to shift toward shorter wavelengths, i.e., laser fragmentation of gold nanoparticles and a decrease in the size of the nanoparticles occur. Figure 6B shows the dependence of the absorption maximum position on the laser pulse energy. It is shown that an increase in the pulse energy to 0.1 mJ (321 mJ/cm^2^) leads to a monotonic change in the absorption maximum position from 525 nm to 539 nm. With a further increase in the pulse energy, the absorption maximum in the gold nanoparticle colloid shifts toward shorter wavelengths and shifts to 521 nm at a pulse energy of 1.5 mJ (4.9 J/cm^2^). In [20], it was shown that the threshold values of laser radiation fluence for the onset of the laser fragmentation process when irradiating aqueous colloids of gold nanoparticles with sizes of several tens of nm with nanosecond laser pulses at a wavelength of 532 nm are in the range of several hundred mJ/cm^2^. A comparison with the results of the present work, where the threshold value for laser fragmentation is 321 mJ/cm^2^, shows that changes in the radiation wavelength and the shape of the irradiated nanoparticles have little effect on the threshold values of the threshold energy density required for nanoparticle fragmentation.

### 3.4. Effect of Laser Pulse Duration on the Characteristics of AuNPs(III)

**Figure 7:**
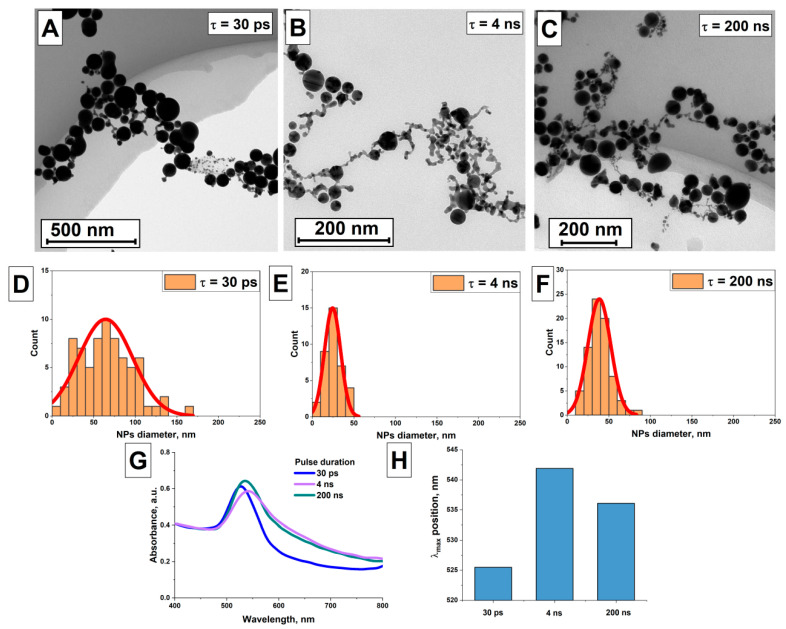
Effect of laser pulse duration on gold particle morphology; (**A**) TEM image of AuNPs(III) particles obtained by irradiating an AuNPs(II) sample with laser radiation (λ = 1064 nm, ε = 50 μJ (159 mJ/cm^2^), t = 8 min) with a pulse duration of 30 ps (scale bar = 500 nm); (**B**) TEM image of AuNPs(III) particles obtained by irradiating an AuNPs(II) sample with laser radiation (λ = 1064 nm, ε = 50 μJ (159 mJ/cm^2^), t = 8 min) with a pulse duration of 4 ns (scale bar = 200 nm); (**C**) TEM image of AuNPs(III) particles obtained by irradiating an AuNPs(II) sample with radiation (λ = 1064 nm, ε = 50 μJ (159 mJ/cm^2^), t = 30 s) with a pulse duration of 200 ns (scale bar is 200 nm); (**D**–**F**) Size distributions of particles reconstructed from TEM images of (**A**–**C**), respectively (the red lines represents the distribution fit); (**G**) Normalized absorption spectra of aqueous colloids of AuNPs(III) particles obtained by irradiating the colloids with radiation with different pulse durations; (**H**) Position of the plasmon resonance peak in the absorption spectra of gold particle colloids depending on the pulse duration.

The effect of laser pulse duration on the morphology of gold particles was studied. Figure 7 shows the results of experiments on irradiating colloids of gold nanoparticles AuNPs(II) with laser pulses for 8 min with a pulse energy of 50 μJ (energy density of 159 mJ/cm^2^) for 30 ps, 4 ns and for 30 s with a pulse energy of 50 μJ (energy density of 159 mJ/cm^2^) for 200 ns pulses. TEM images of gold nanoparticles were obtained for each pulse duration (Figure 7A–C). As a result of irradiation, both elongated remelted particles and large spherical gold particles are present in the colloid. For each TEM image of nanoparticles, the particle size distribution was reconstructed (Figure 7D–F). The obtained distributions are monomodal, with the particle distribution maxima for pulse durations of 30 ps, 4 ns, and 200 ns occurring at sizes of 63 nm, 24 nm, and 40 nm, respectively. The absorption spectrum of the resulting gold nanoparticle colloids was analyzed (Figure 7G). It was shown that the absorption spectra of the nanoparticle colloids obtained using laser radiation with different pulse durations are characterized by different absorption peak positions. Figure 7H shows a histogram with the position of the absorption maximum depending on the pulse duration. For gold nanoparticle colloids obtained by irradiation with laser pulses of 30 ps duration, the absorption maximum is at 526 nm, for a pulse duration of 4 ns the absorption maximum is at 542 nm and for pulses of 200 ns duration the absorption maximum is at 536 nm. It is worth noting that the absorption spectrum of the nanoparticle colloid obtained by irradiation with picosecond laser pulses exhibits lower absorption in the long-wavelength region. This may indicate a lower concentration of elongated nanoparticles in the colloid, compared to other samples. This is also evident in the TEM images. In addition, the plasmon resonance peak of gold nanoparticles obtained by irradiation with picosecond pulses is located at approximately 524 nm. The absorption spectrum of these nanoparticles does not exhibit any characteristic features of large particles, such as greater absorption in the red region, due to increased scattering. On the other hand, the sizes of gold nanoparticles obtained by irradiating gold nanochain colloids with 200 ns pulses according to the specified distribution have larger sizes than those obtained using 4 ns pulses. However, the plasmon resonance peak in the absorption spectrum is located at 535 nm in the former case, and at 541 nm in the latter. These discrepancies with previous results can be explained, on the one hand, by the low concentration of the large particle fraction, as well as by the aggregation and rapid sedimentation of large particles in the colloid during absorption spectrum measurement. On the other hand, the TEM images presented, as well as the absorption spectra of the colloids obtained with different pulse duration, show that the number of gold nanochains is lowest for picosecond pulses. This indicates the most efficient conversion of gold nanochains into submicron spheres, which is also confirmed by the larger particle sizes. As a result, a small fraction of large particles forms in the colloid, which does not significantly contribute to the colloid’s absorption spectrum. The conversion efficiency of gold nanochains is lowest for pulses of 4 ns duration. That is, with the chosen synthesis parameters, the number of remelted spheres and nanochains is sufficient to observe a shift in the plasmon resonance in the absorption spectrum.

Thus, it has been demonstrated that, all other factors being equal (laser radiation energy density, irradiation time, nanoparticle concentration), the use of picosecond laser pulses allows the synthesis of larger submicron gold particles compared to nanosecond pulses. This fact may indicate fundamental differences in the interaction of laser pulses of different duration with nanoparticles. Specifically, nanosecond pulses may partially disrupt gold nanochains, preventing them from subsequently fusing with each other due to surface tension forces in the molten material. In contrast, picosecond pulses result in local melting of the material within the gold nanochain, sufficient to initiate the synthesis of large spherical particles without disrupting the nanochains.

### 3.5. The Influence of Gold NP Concentration in the Initial Colloid (AuNPs(II)) on the Characteristics of AuNPs(III)

**Figure 8:**
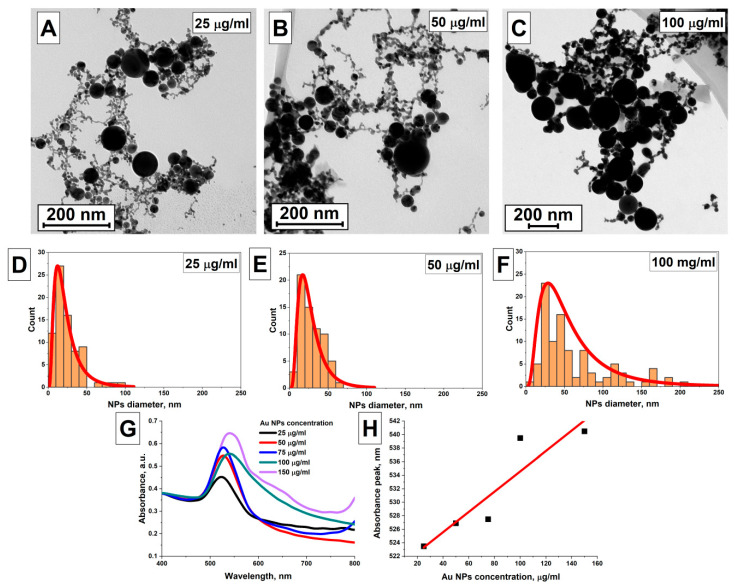
Effect of particle concentration in the irradiated colloid on the morphology of gold nanoparticles; (**A**) TEM image of AuNPs(III) particles obtained by irradiating an AuNPs(II) sample with radiation (λ = 1064 nm, ε = 50 μJ (159 mJ/cm^2^), t = 8 min) with a concentration of 25 μg/mL (scale mark is 200 nm); (**B**) TEM image of AuNPs(III) particles obtained by irradiating an AuNPs(II) sample with radiation (λ = 1064 nm, ε = 50 μJ (159 mJ/cm^2^), t = 8 min) with a concentration of 50 μg/mL (scale mark is 200 nm); (**C**) TEM image of AuNPs(III) particles obtained by irradiating an AuNPs(II) sample with radiation (λ = 1064 nm, ε = 50 μJ (159 mJ/cm^2^), t = 8 min) with a concentration of 100 μg/mL (scale bar is 200 nm); (**D**), (**E**), (**F**) Nanoparticle size distributions reconstructed from TEM images (**A**), (**B**) and (**C**), respectively (the red lines represents the distribution fit); (**G**) Normalized absorption spectra of aqueous colloids of AuNPs(III) particles obtained by irradiating colloids of particles with different concentrations; (**H**) Position of the plasmon resonance peak in the absorption spectra of gold nanoparticle colloids as a function of the concentration of nanoparticles in the irradiated colloid (the red line represents the data fit).

The influence of the concentration of nanoparticles in the irradiated AuNPs(II) sample on the morphology of AuNPs(III) gold particles was established. The irradiation of AuNPs(II) samples with concentrations of 25 μg/mL, 50 μg/mL, 75 μg/mL, 100 μg/mL, 150 μg/mL was carried out using Nd:YAG laser radiation (λ = 1064 nm, ε = 50 μJ (159 mJ/cm^2^), t = 8 min). Figure 8A–C show TEM images of AuNPs(III) gold samples obtained by irradiating AuNPs(II) gold with concentrations of 25 μg/mL, 50 μg/mL and 100 μg/mL. The TEM images of samples show that the colloids contain both elongated gold nanoparticles of the AuNPs(II) sample and spherical gold particles AuNPs(III). The sizes of large gold particles in the TEM images vary from 50 to 150 nm. Figure 8D–F show the size distributions of gold particles reconstructed from the TEM images. It was found that for a sample of particles with a concentration of 25 μg/mL, the distribution maximum is at sizes of 15–20 nm, the half-width of the distribution is 21 nm, in addition, it is clear that large particles with sizes from 60 to 100 nm are also present in the distribution, Figure 8D. In the distribution of gold particles with a concentration of 50 μg/mL, the distribution maximum is located at 18 nm, the half-width of the distribution is 28 nm, Figure 8E. The maximum of the particle size distribution in a sample of gold particles with a concentration of 100 μg/mL is located at 29 nm, the half-width of the distribution is 54 nm, the distribution contains gold particles with sizes from 50 to 200 nm, Figure 8F. The absorption spectra of colloids of gold particles AuNPs(III) with different particle concentrations were studied, Figure 8G. It is shown that an increase in the concentration of particles in the irradiated colloid from 25 μg/mL to 150 μg/mL leads to an increase in the amplitude of the plasmon resonance peak and its shift towards longer wavelengths. An increase in absorption in the red region of the spectrum is also observed in the absorption spectra of colloids with concentrations of 100 μg/mL and 150 μg/mL. Figure 8H shows the dependence of the absorption maximum position of gold particle colloids on concentration. It was shown that with an increase in the concentration of particles, the position of the absorption peak in the colloid after irradiation monotonically shifts from 523 nm at a concentration of 25 μg/mL to 540 nm at a concentration of 150 μg/mL.

In [27,54], submicron spherical gold particles were obtained by irradiating colloids with defocused laser radiation. It was shown that the key factor in the process of synthesis of submicron gold particles is the control of nanoparticle aggregation. This, in turn, is influenced by ligands, namely citrate and NaCl solution. Moreover, the study showed that the agglomeration of the initial nanoparticles is controlled not only by the ligands, but is also induced by laser radiation, which removes ligand molecules from the surface of the initial nanoparticles, leading to their aggregation and subsequent aggregation. Since the rate of nanoparticle aggregation directly depends on the concentration, with increasing concentration in the colloid, there will be longer chains of gold nanoparticles, their number in the colloid will be greater. This, when they are irradiated, will lead to an increase in the size of the nanoparticles.

**Figure 9:**
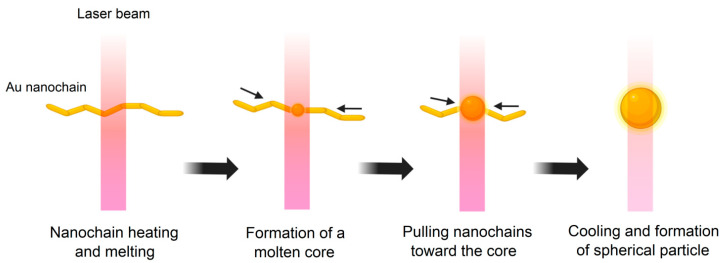
Schematic representation of the mechanism of formation of spherical submicron gold particles AuNPs(III) upon irradiation of a colloid of gold nanochains AuNPs(II).

Taking into account all the results obtained above, the proposed mechanism for the formation of submicron spherical gold particles upon irradiation of gold nanochain colloids is shown in Figure 9. Initially, the laser pulse interacts with the nanochain material, causing it to heat and melt. In [55,56], it was shown that melting and change in shape of elongated gold nanoparticles occurs at temperatures significantly lower than the melting temperature of gold, which is due to the effect of surface melting of particles, which in turn allows for the shape of the chains to be changed without compromising their structural integrity. Subsequent local melting of the gold nanochain surface results in the formation of a spherical molten gold core in one segment of the nanochain. Due to the surface tension, this molten core begins to attract the chain toward the center. It is important to note that the laser pulse energy does not exceed the evaporation energy of the nanochain material. Otherwise, a decrease in particle size would be observed due to laser fragmentation. After the pulse ends, the molten gold core begins to cool, forming a larger spherical gold particle. In [54], it has been shown that the primary mechanism for the formation of submicron gold particles is the agglomeration of the initial nanoparticles, which in turn is regulated by the citrate concentration in the solution. In our case, the key feature is the irradiation of already aggregated gold nanochains, which were previously obtained by irradiating a colloid of spherical gold particles with second harmonic radiation. This eliminates the need for chemicals and ligands in the synthesis process.

As the colloid irradiation time increases, a gradual conversion of all gold nanochains into submicron spheres occurs. The rate of formation of submicron particles as a result of melting is highest in the first minutes of colloid irradiation, followed by a gradual slowdown in the rate of formation of larger particles (Figure 5). This decreasing efficiency is due to the lack of radiation absorption centers and the formation of nanoparticle melts. Additionally, larger gold particles begin to effectively scatter radiation within the colloid, resulting in an energy density insufficient for melting. Varying the laser pulse energy also allows for the control of the size of the resulting particles. The highest efficiency is achieved using pulses with an energy density slightly below the threshold energy density for laser fragmentation of nanoparticles. Increasing the nanoparticle concentration predictably leads to an increase in the final size of the submicron particles in the colloid, due to the fact that the irradiated colloid contains a larger number of nanochains, which act as centers for the formation and absorption of radiation. The most interesting result was obtained when studying the effect of laser pulse duration on the size of the resulting particles. Picosecond pulses prove to be the most effective; for such pulses, the most efficient conversion of chains into gold spheres is observed, and the size of the resulting spherical particles is also the largest, all other parameters being equal. Presumably, the observed results are associated with the effect of local surface melting of the nanoparticle material. Nanosecond pulses can lead to undesirable thermal effects that can contribute to the rupture of nanochains, limiting the final size of spherical particles when irradiating colloids.

Thus, this study demonstrated that the sequential processes of laser ablation of a target in liquid, laser-induced aggregation, and laser irradiation of gold nanoparticle colloids can produce particles with submicron sizes (up to 200 nm) from nanoparticles with initial dimensions of 20 nm. The key laser radiation parameters that influence the process of submicron particle synthesis were investigated: the irradiation time of the colloids, the pulse energy, and the pulse duration. The effect of nanoparticle concentration in the initial colloid on final particle sizes was also examined. As noted previously, the advantage of the method demonstrated in this study is the ability to produce spherical submicron gold particles without the use of external chemical reagents. However, one of the drawbacks of the presented synthesis method may be the low yield of submicron-sized particles. We have shown the existence of optimal parameters of laser radiation (irradiation duration no higher than 8–10 min, pulse energy/fluence up to threshold values of laser fragmentation of 100–300 mJ/cm^2^, picosecond pulse durations) and concentration (proportional dependence of sizes) in the process of melting gold nanochains allows the efficiency of the yield of submicron particles to significantly increase. At this stage, the search for optimal parameters and experimental configurations to increase the yield of particles at the stage of laser ablation and laser synthesis of nanochains will be the subject of further research.

## 4. Conclusions

The results of this study demonstrate that sequential processes of laser ablation of a target in liquid, laser-induced aggregation with nanochain formation, and laser irradiation of colloidal gold nanochains produce spherical gold particles of submicron sizes (100–200 nm). It is shown that the final size of gold particles obtained through the three-stage synthesis process is influenced by the irradiation time during the laser melting stage, the initial concentration of nanoparticles, the energy and duration of the laser pulse. The developed method allows for an expansion of the size range of nanoparticles obtained by physical methods involving laser radiation without the use of external reagents, thereby simplifying the nanoparticle synthesis procedure and maintaining the purity of the colloids for their subsequent application and use.

## Data Availability

The data presented in this study are available on request from the corresponding author (Order of the Director of the Institute).
